# Experimental evidence of bark beetle adaptation to a fungal symbiont

**DOI:** 10.1002/ece3.1772

**Published:** 2015-10-19

**Authors:** Ryan R. Bracewell, Diana L. Six

**Affiliations:** ^1^ Department of Ecosystem and Conservation SciencesThe University of Montana32 Campus DriveMissoulaMontana59812

**Keywords:** *Dendroctonus*, *Entomocorticium*, mutualism, mycangia, symbiosis

## Abstract

The importance of symbiotic microbes to insects cannot be overstated; however, we have a poor understanding of the evolutionary processes that shape most insect–microbe interactions. Many bark beetle (Coleoptera: Curculionidae, Scolytinae) species are involved in what have been described as obligate mutualisms with symbiotic fungi. Beetles benefit through supplementing their nutrient‐poor diet with fungi and the fungi benefit through gaining transportation to resources. However, only a few beetle–fungal symbioses have been experimentally manipulated to test whether the relationship is obligate. Furthermore, none have tested for adaptation of beetles to their specific symbionts, one of the requirements for coevolution. We experimentally manipulated the western pine beetle–fungus symbiosis to determine whether the beetle is obligately dependent upon fungi and to test for fine‐scale adaptation of the beetle to one of its symbiotic fungi, *Entomocorticium* sp. B. We reared beetles from a single population with either a natal isolate of *E*. sp. B (isolated from the same population from which the beetles originated), a non‐natal isolate (a genetically divergent isolate from a geographically distant beetle population), or with no fungi. We found that fungi were crucial for the successful development of western pine beetles. We also found no significant difference in the effects of the natal and non‐natal isolate on beetle fitness parameters. However, brood adult beetles failed to incorporate the non‐natal fungus into their fungal transport structure (mycangium) indicating adaption by the beetle to particular genotypes of symbiotic fungi. Our results suggest that beetle–fungus mutualisms and symbiont fidelity may be maintained via an undescribed recognition mechanism of the beetles for particular symbionts that may promote particular associations through time.

## Introduction

Many insects are involved in symbiotic associations with microbes that provide nutrition crucial for insect survival (Mueller et al. 2005; Moran 2007). In obligate endosymbioses where the symbiont is transferred vertically from parent to offspring, both theoretical and empirical studies have demonstrated the relative ease at which coevolution (reciprocal adaptation) and co‐cladogenesis can occur (Clark et al. 2000; Conord et al. 2008; Moran and Bennett 2014). However, a large number of insect–microbe symbioses, and particularly insect–fungal symbioses, are ectosymbioses, where symbiont transfer can be imperfectly vertical or even horizontal (Mueller et al. 2005). In such systems, coevolution and/or co‐cladogenesis has been regarded as less likely to occur due to the potential for swapping and invasion. Despite this assumption, numerous studies have now observed strong fidelity among hosts and symbionts in several ectosymbioses (Alamouti et al. 2011; Mehdiabadi et al. 2012; Seal and Mueller 2014). However, the mechanisms that maintain fidelity as well as the occurrence of coevolution remain severely understudied in most ectosymbiotic systems.

Bark beetles (Coleoptera: Curculionidae, Scolytinae) are some of the most ecologically and economically important forest insects and many are involved in tightly linked ectosymbioses with fungi (Paine et al. 1997; Harrington 2005; Six 2012). These symbioses remain understudied in many important aspects including how dependent the partners are upon one another, and whether hosts and symbionts exhibit coevolution or codiversification (Six and Paine 1999; Six 2012). Some bark beetle symbioses exhibit characteristics that imply coevolution. A number of bark beetle species have evolved specialized exoskeletal structures, called mycangia, that aid in transporting their fungal symbionts between host trees. The fungal symbionts also exhibit adaptations to their hosts including the production of sticky spores that are specialized for insect transport (Upadhyay 1981; Jacobs and Wingfield 2001; Hsiau and Harrington 2003). These symbioses are generally considered mutualisms because the mycangial fungi gain transportation to host trees while, in return, the fungi provide nutritional benefits to the developing insect (Coppedge et al. 1995; Ayres et al. 2000; Bleiker and Six 2007). Most mycangium‐bearing bark beetle–fungal symbioses are also considered obligate, although very few have been experimentally tested in this regard. In part, this has been due to the difficulty of experimentally manipulating the symbiosis.

**Figure 1:**
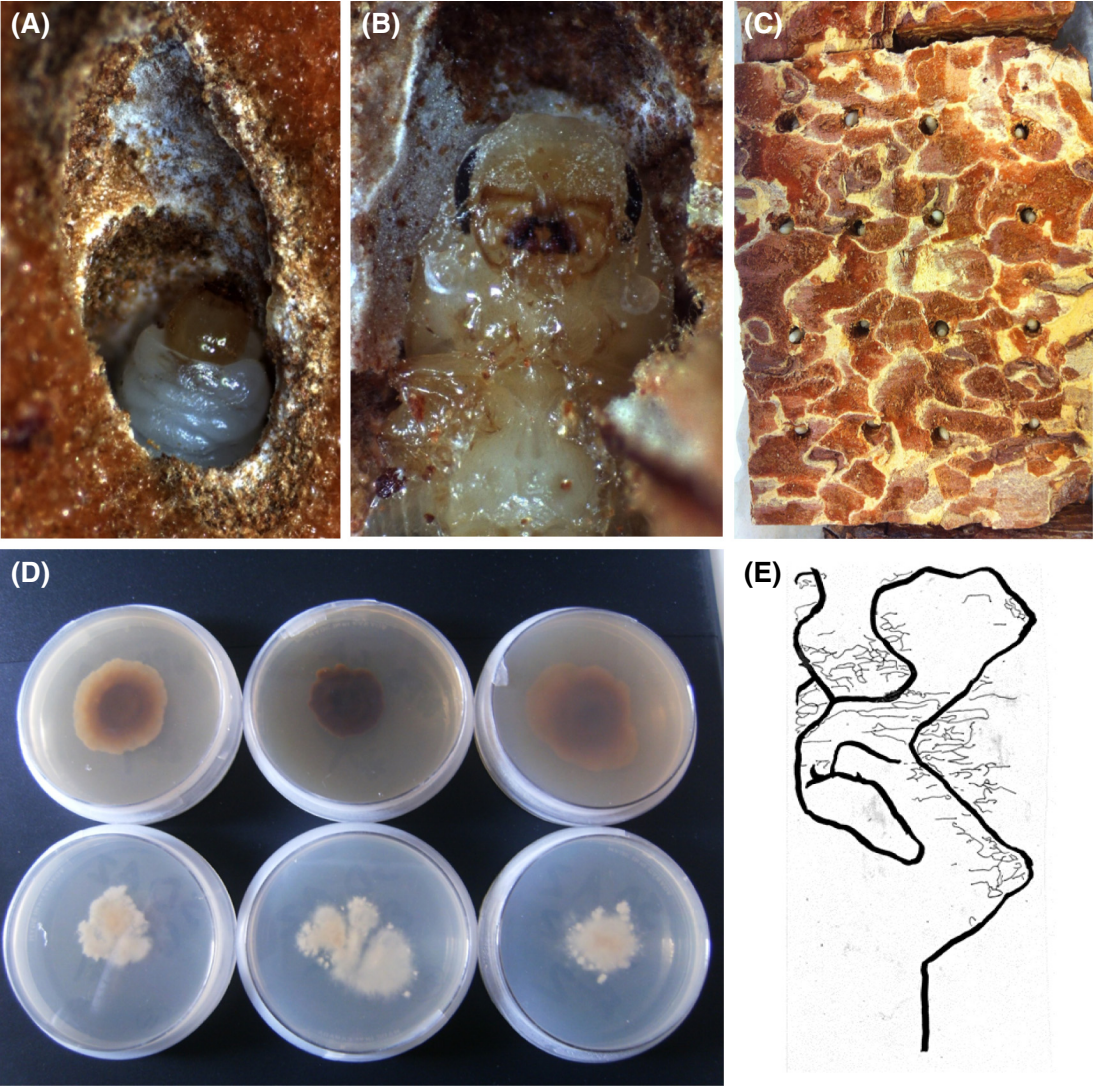
(A) Western pine beetle feed heavily on symbiotic fungi while developing in ponderosa pine bark (fungi seen as white mats in larval tunnel). (B) During pupation in the bark, fungal spores line the pupal chamber for incorporation into the mycangia after metamorphosis. (C) For our experiments, we created pseudo‐pupal chambers in ponderosa pine bark to rear aposymbiotic adult western pine beetles. Each chamber contains a pupa. (D) Two genetically, geographically, and phenotypically distinct isolates of *Entomocorticium* sp. B (haplotype A = natal, shown above, haplotype C = non‐natal, shown below) were used to test for adaptation of beetles to particular fungi. e) Example of transparency tracings of typical parent tunnels (thick lines) and larval tunnels (thin lines) of a western pine beetle gallery from one of the fungus treatments.

The western pine beetle (*Dendroctonus brevicomis* LeConte) symbiosis is a powerful system to address questions of coevolution and codiversification in ectosymbioses, in general, and beetle–fungus symbioses, in particular. This symbiosis involves a beetle with two symbiotic fungal partners, *Entomocorticium* sp. B (Basidiomycota) and *Ceratocystiopsis brevicomi* (Ascomycota) (Whitney and Cobb 1972; Paine and Birch 1983; Hsiau and Harrington 1997, 2003), that show remarkable fidelity with their host across its entire range (Bracewell and Six 2014). *Entomocorticium* sp. B and *C. brevicomi* have also never been found outside of the western pine beetle symbiosis. The two fungi are carried in a prothoracic mycangium found only in females. During tree colonization, they inoculate the tree with their symbiotic fungi and oviposit in the tree's phloem layer. Larval feeding and development initially occurs in the phloem where the developing larvae feed on a combination of fungi and phloem. However, at about the second instar, larvae transition from the more nutrient‐rich phloem to the nutrient‐poor bark (Miller and Keen 1960) (Fig. 1A and B). This transition is hypothesized to be mediated by the symbiotic fungi, of which one species, *E*. sp. B, may be particularly important given that *Entomocorticium* species are cellulolytic and can grow not just on phloem but also on bark (Valiev et al. 2009).

The western pine beetle is restricted to ponderosa pine (*Pinus ponderosa* Douglas) across most of its range. Genetic evidence indicates the beetle is actually two cryptic species that are geographically isolated on two subspecies of ponderosa pine (Kelley et al. 1999). Mitochondrial DNA sequence divergence suggests these cryptic species of beetle have been isolated for a few million years (Kelley et al. 1999). Palynological and molecular data for the tree suggest that the tree subspecies formed in glacial refugia during the Pleistocene (Conkle and Critchfield 1988; Betancourt et al. 1990; Latta and Mitton 1999; Potter et al. 2015). There is also evidence of genetic divergence within *E*. sp. B that corresponds to patterns of divergence in both the host tree and insect (Bracewell and Six 2014). Three distinct haplotypes of *E*. sp. B have been identified, and haplotypes A and B co‐occur and are found only in beetle populations in the westernmost portion of the distribution (CA, OR, WA, ID, MT, and BC, Canada), while haplotype C occurs exclusively in the southwestern United States (CO, UT, AZ, NV, NM).

A powerful way to test for dependency and adaptation is to conduct symbiont removals and experimental swapping of symbionts (de Fine Licht et al. 2007; Seal and Mueller 2014). Although the western pine beetle–fungus symbiosis has been described as an obligate mutualism, to date there have been no manipulative experiments to test this hypothesis, nor whether adaptation of the beetle to particular fungi has occurred. Here, we focus our investigation on one symbiont of this beetle, *E*. sp. B, because this partner is thought to be the superior symbiont in this system for supporting beetle nutrition. Adult beetles captured carrying *E*. sp. B tend to be, on average, larger than those developing with *C. brevicomi*, suggesting that developing larvae gain more nutrition while feeding on this fungus (Bracewell and Six 2014). *Entomocorticium* sp. B is also more prevalent than *C. brevicomi*, indicating it may play a dominant role in the symbiosis. Further, the genetic differences found between *E*. sp. B haplotypes indicate that phenotypic differences may occur among the haplotypes that could alter the symbiosis.

The objectives of this study were to determine whether (1) mutualistic fungi, particularly *E*. sp. B, are crucial for western pine beetle development and (2) whether we could detect evidence of adaptation by the beetle to specific isolates of *E*. sp. B. We did this by rearing beetles from one cryptic species with their normal haplotype of *E*. sp. B (haplotype A, designated here as natal), with a haplotype associated with the other cryptic beetle species (haplotype C, designated here as non‐natal,), or with no fungi. We then characterized the effect of these three treatments on beetle development and fitness.

## Methods

### Generating aposymbiotic adult beetles

Live western pine beetles were collected near Missoula MT (46°49′ N, 114°08′ W) in June 2012 using Lindgren funnel traps baited with chemical attractants (Synergy Semiochemical Corp., Burnaby, BC, Canada; part P130, western pine beetle trap lure). All adult beetles were surface‐sterilized (30 sec in 70% EtOH) to remove potentially antagonistic fungi which can be carried externally and hamper laboratory rearing. Sex was then determined by the presence of the mycangial swelling on the pronotum of females and tubercles on the frons of males (Wood 1982). Males and females were then paired in sections of a freshly cut ponderosa pine felled at The University of Montana Lubrecht Experimental Forest (46°53′ N, 113°28′ W). Detailed methods for rearing bark beetles are described elsewhere (Bracewell et al. 2011). Specific to this study, a total of 15 sections of ponderosa pine (~33 cm in length) were each infested with 10–13 beetle pairs. To produce large numbers of pupae, tree sections containing beetles were stored at room temperature (~21°C) for 40 day allowing most to reach the pupal stage (Miller and Keen 1960). Pupae were collected from the sections by removing the bark which was then fractured to expose the pupal chambers. Pupae were then removed and placed in Petri dishes lined with filter paper moistened with distilled water.

Larvae void their guts prior to pupation but may still carry microbes including fungi on their exoskeletons. Therefore, we surface‐sterilized the pupae using a series of three short EtOH washes conducted over 3 days. Washes consisted of placing pupae for 10 sec in 70% EtOH, before quickly dipping them in distilled water and transferring them to a Petri dish. We then placed surface‐sterilized pupae into pseudo‐pupal chambers constructed from fresh ponderosa pine bark (Fig. 1C). To mimic a western pine beetle pupal chamber, 8 × 8 × 3 cm pieces of ponderosa pine bark were cut from a tree and small holes were drilled into the bark piece (Fig. 1C). To maintain humidity for the developing insect, bark pieces containing pupae were placed into plastic containers floating in a bath of distilled water in air‐tight rearing containers. The rearing containers were maintained at room temperature (~21°C) and pupae allowed to develop into adults.

Approximately 350 pupae were placed into pseudo‐pupal chambers. Rearing containers were checked daily and any eclosed and putatively “fungus‐free” adults were collected and placed into sterile Petri dishes and held at ~4°C. Due to the difficulties of manipulating small insects during a sensitive life stage, a large number of pupae were processed to ensure enough individuals survived for use in the experiment. To confirm that adult beetles did not have fungi in their mycangia following rearing and surface sterilization, we attempted to isolate fungi from the mycangia of nine females (14% of all females), all of which were negative for fungi. Methods used to isolate mycangial fungi from western pine beetles are detailed in Bracewell and Six (2014).

### Propagating fungal symbionts

Two isolates of fungi were used in this study. The natal isolate (MI22) was originally isolated from a beetle collected near Missoula MT (46°49′ N, 114°08′ W). The non‐natal isolate (RO10) was originally isolated from an individual of the other cryptic western pine beetle species collected near Ruidoso NM (33°28′ N, 105°44′ W) (Bracewell and Six 2014). Both isolates have typical morphology and display the same growth patterns on MEA of the fungal populations from which they were isolated (Bracewell and Six 2014). Although they are both currently considered *E*. sp. B, they are genetically divergent; MI22 has been identified as haplotype A and RO10 as haplotype C (Bracewell and Six 2014). Pairwise distance between A and C haplotypes (p‐distance) is 0.004 over the ITS2‐LSU region (Bracewell and Six 2014). Haplotype A and haplotype C are also visually distinct when grown on 2% MEA (Fig. 1D). The isolates were grown on 2% MEA for ~3 weeks prior to experimental manipulation to ensure the mycelia were in the active growth stage.

### Manipulating the symbiosis

One ponderosa pine was felled at Lubrecht Experimental Forest (46°53′ N, 113°28′ W) in August 2012. The tree was first cut crosswise into 33‐cm sections. Then, each section was quartered lengthwise resulting in a total of 198 cm^2^ of phloem/bark for each beetle pair per replicate. Tree sections were coated in paraffin wax along the four cut edges to help maintain natural levels of moisture.

Due to slight differences in phloem thickness among sections which could influence total brood production, treatments (natal, non‐natal, and no fungus) were randomly assigned to the tree sections. Seventeen tree sections (replicates) per treatment were assigned to each of the three treatments at the start of the experiment. To establish the fungus in a section, one 4‐mm‐diameter plug of agar was taken from an MEA plate containing either the natal or the non‐natal fungus. No fungus treatments received a plug of MEA. Each plug was smeared inside a hole drilled into the phloem layer at the base of the tree section. Trials conducted prior to our experiment indicated that 7 days was adequate for both the natal and the non‐natal fungus to establish in the tree phloem. Therefore, after inserting the agar plug, all tree sections were held for 7 days. Surface‐sterilized female/male pairs were then inserted into the same drill hole (female first), and a piece of wire screen was fixed over the hole to prevent escape. Each tree section was then placed in a rearing container and monitored daily for emergence of offspring. Tree sections were maintained at room temperature (~21°C) under natural light conditions.

To assess the effect of a particular treatment on brood production and the resulting fitness of offspring, we recorded when each brood beetle emerged, their sex, and their pronotum width. Pronotum width is a proxy measure for overall beetle size (Bentz et al. 2001) and size is positively correlated with offspring production (Honek 1993). To estimate the pronotum width, digital images were taken using a Leica EZ4D stereomicroscope with built‐in 3‐megapixel camera. To confirm fungal growth in the tree sections, we visually inspected all replicates after completion of the experiment. To confirm we were able to re‐establish the symbiosis by inoculating the tree sections with fungal isolates, we isolated fungi from the mycangia of a subset of brood females from each replicate soon after they emerged into the rearing containers. We also confirmed which fungal isolate (MI22 or RO10) was recovered from each mycangium by sequencing the ITS2‐LSU region using the primer pair ITS3 and LR3 (Vilgalys and Hester 1990; White et al. 1990). Methods used to isolate the mycangial fungi and to perform PCR and DNA sequencing are described in Bracewell and Six (2014). Sequences were compared to reference sequences for MI22 and RO10 (Genbank accessions KJ620521 and KJ620518).

To further investigate the effects of the three treatments on western pine beetle brood production, development, and fitness, parent and larval tunnels were measured after brood emergence was complete. We traced the parent galleries and all larval tunnels on transparency sheets (Fig. 1E). Parent gallery length was estimated as the sum of all tunnels created by parent beetles per section. Due to the network of tunnels created by larvae, and the state of decay of the samples after completion of the experiment, it was impossible to estimate the number of larvae or egg niches produced by each beetle pair. However, we could measure total larval tunnel length which allowed us to determine whether larvae from the three treatments fed differentially in the phloem layer (an indication of differential nutrient availability due to the presence/absence of symbiotic fungi). Here, total larval tunnel length was calculated for each replicate as the sum of larval tunnel distances divided by the total gallery length of the parents. In other studies, a positive correlation has been found between parent gallery length and reproductive output (Amman 1972; Anderbrant 1990). Therefore, this metric provides an estimate of larval feeding differences that takes into account the different quantities of larvae found in galleries of differing lengths.

### Statistical analysis

All analyses were conducted using the statistical package R v. 3.1.2 (R Development Core Team, 2011). To investigate the effect of the fungal treatments on development time and on the size of brood beetles, and because both development time and beetle size were found to be normally distributed, we fit linear mixed models (LMMs) using the *nlme* package in R (Pinheiro et al. 2015). For beetle size comparisons, we analyzed the male and female data separately, because females are on average larger than males (Foelker and Hofstetter 2014). We treated each replicate as a random effect in the model to account for nonindependence of brood beetles within replicates. *Post hoc* pairwise comparisons between the three treatments were performed using Tukey's HSD (honestly significance difference) tests in the R *multcomp* package (Hothorn et al. 2014). To test for the influence of fungal treatment on total length of parent gallery, our standardized total larval tunneling length measure, number of offspring, and proportion of brood that were female, we fit generalized linear models (GLMs) with the *glm* package in R and specified appropriate error distributions for each response variable. Significance of the fixed effects in the model was determined using Wald chi‐square tests, and pairwise comparisons between treatments were performed using Tukey's HSD tests. Total larval tunnel lengths and length of parent galleries were found to be normally distributed and were modeled with Gaussian distributions. The number of offspring was count data and so was modeled using a poisson distribution. The proportion of females produced was found to be overdispersed (more variance than expected) and thus modeled using a quasi‐binomial distribution. For all models, adequate model fit was determined by evaluating the residual deviance.

## Results

The experiment ran for 312 days and was stopped when no brood beetles emerged from any replicate for >14 days. At the completion of the experiment, 14, 12, and 13 replicates per treatment (natal, non‐natal, and no fungus, respectively) were considered for analyses. Criteria for inclusion in analysis were that parent gallery length was >0 cm (indicating successful pairing and tunneling by the parents).

A total of 742 brood adult beetles were recovered from rearing containers. A subset of brood females from each replicate (when present) were used to isolate fungi from their mycangia to confirm that experimentally manipulated fungi were successfully transferred to the gallery of the insect and subsequently acquired in brood adult mycangia. We isolated fungi from the mycangia of 1 to 14 beetles from all replicates that produced females and in total isolated fungi from 132 individuals (Appendix 1). Replicates where we were unable to attempt fungal isolation from a female (*n* = 3 natal, 11 no fungus, 2 non‐natal) were kept in their respective treatment category. We sequenced the ITS2‐LSU regions of three isolates from the mycangia of beetles from three replicates each of the natal fungus treatment and the non‐natal fungus treatment (only three replicates had fungi), and one isolate from the only replicate of the no fungus treatment that was positive for mycangial fungi. All of the isolates possessed DNA sequences identical to haplotype A, indicating that there were instances in the non‐natal and no fungus treatments where surface sterilization of the pupae was unsuccessful. After removing these replicates from further analyses, we were left with, 14, 9, and 12 replicates per treatment (natal, non‐natal, and no fungus, respectively). For the natal fungus treatment, we recovered natal fungi from 73% of replicates (eight of 11 replicates, 31 of 49 beetles, mean = 4 females isolated per replicate). In contrast, we were unable to recover the non‐natal fungus from a single mycangium of brood females (0 of 7 replicates, 0 of 65 beetles, mean = 8 females isolated per replicate) (*χ* ^2^ (1, *N *=* *114) = 56.48, *P *<* *0.0001). All tree sections in the natal and non‐natal treatments showed evidence of *E*. sp. B growth in the phloem in both the parent gallery and the larval tunnels.

**Figure 2:**
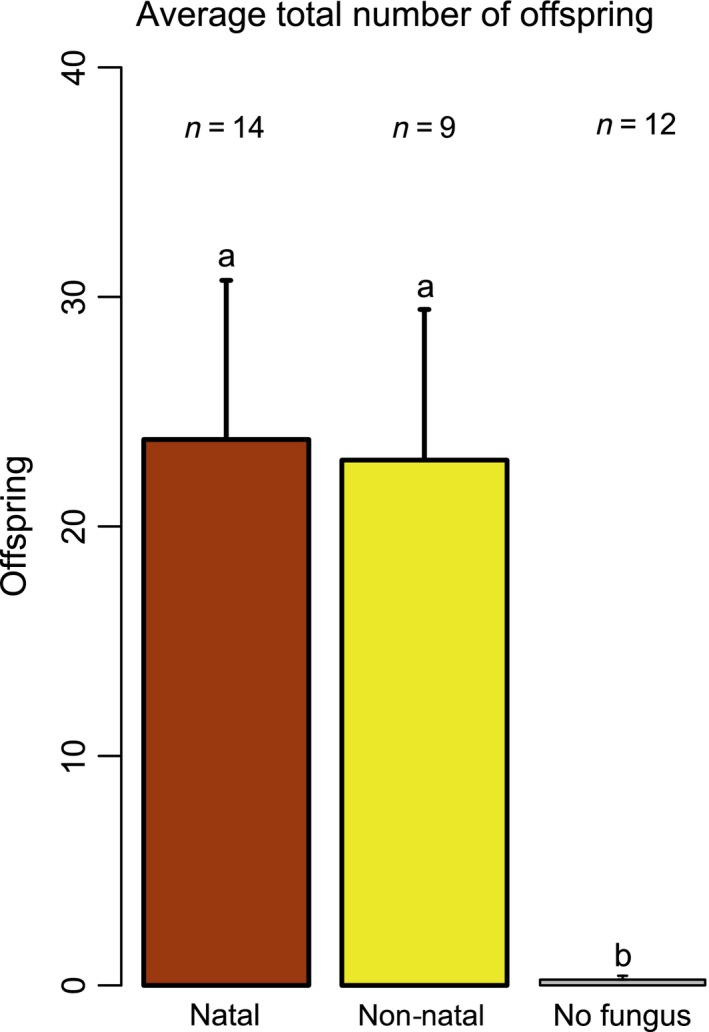
Average total number of offspring (error bars = SEM) from the natal, non‐natal, and no fungus treatments. The number of male/female pairs per treatment are denoted above their respective bar. Bars with the same letter are not statistically significantly different from one another (Tukey HSD test).

We found no significant differences in parent gallery length among the three treatments (Tables 1, 2) suggesting that in terms of gallery construction, the parent beetles were not affected. There was evidence of larval tunnels (and therefore, oviposition, egg hatch, and larval feeding), in all treatments, and no significant differences were found in the amount of larval tunneling among treatments (Tables 1, 2). Although there was no evidence of a decrease in the level of reproductive input from parent adults, or alteration in tunneling distance by larvae, there was a highly significant difference in the total number of offspring produced across treatments (Tables 1, 2). There was a near absence of adult offspring in the no fungus treatment, while the natal and non‐natal treatments produced adult offspring but did not differ significantly in number (Fig. 2). Examination of larval tunnels in the no fungus treatment indicated that nearly all larvae perished prior to tunneling into the bark. The proportion females produced was not statistically different between the natal and non‐natal treatments (Tables 1, 2).

**Table 1:** Mean (SE) of parent and larval gallery lengths, total number of offspring produced, and proportion of female to male offspring produced by western pine beetle developing with no fungi or fungal treatments. Values in the same column followed by the same letter are not significantly different (Tukey's HSD test, *α *= 0.05)

Treatment	*N*	Parent gallery length (cm)	Larval tunneling (cm)	Total offspring	Proportion female
Natal	14	65.96 (5.94) a	1.53 (0.31) a	23.79 (6.93) a	0.437 (0.19) a
Non‐natal	9	85.86 (13.10) a	1.46 (0.33) a	22.89 (6.56) a	0.521 (0.19) a
No fungus	12	62.86 (6.77) a	2.05 (0.44) a	0.25 (0.17) b	a

a Unable to estimate due to too few individuals.

**Table 2:** Results from GLM analysis of the influence of fungal treatment (natal, non‐natal, no fungus) on four measures of western pine beetle reproductive success (Response variable)

Response variable	Factor	Wald chi‐square	df	*P‐*value
Parent gallery length	Intercept	85.5	1	<0.0001
	Treatment	4	2	0.14
Larval tunneling	Intercept	20.8	1	<0.0001
	Treatment	1.5	2	0.47
Total offspring	Intercept	3344.4	1	<0.0001
	Treatment	61.7	2	<0.0001
Proportion female	Intercept	3.4	1	0.066
	Treatment	3.2	1	0.074

**Figure 3:**
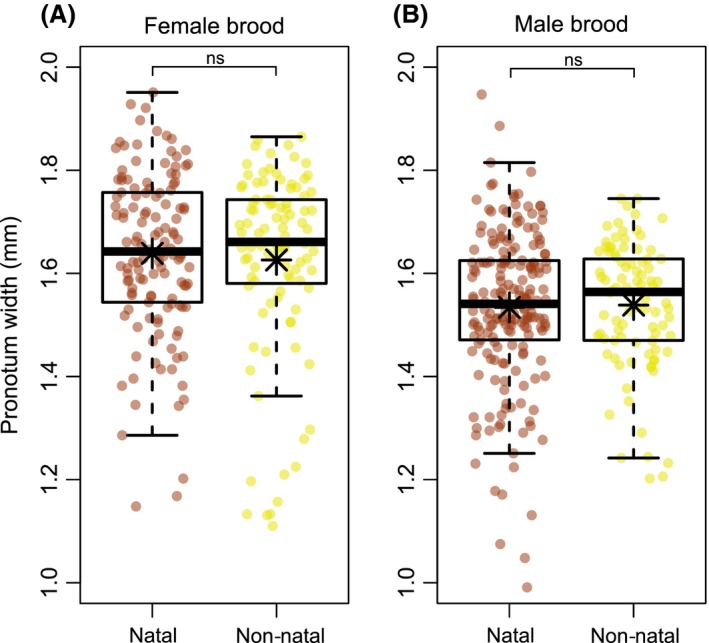
Comparison of size (pronotum width) of brood A) females and B) males from the natal and non‐natal fungal treatments. Each pronotum measure is represented by a point on their respective boxplot and the mean size per treatment is denoted with an asterisk. Not significant = ns.

When comparing the relative size of brood from the natal and non‐natal treatments, and with respect to brood females, we did not find a relationship between adult size and development time (*F*_1,209_ = 0.330, *P *=* *0.567). There was also no effect of fungal treatment (*F*_1,16_ = 0.372, *P *=* *0.550) (Fig. 3A), and no interaction between development time and fungal treatment (*F*_1,209_ = 2.029, *P *=* *0.156). Results from size comparisons of brood males were similar to that of females. There was no significant relationship between the size of male beetles and their development time (*F*_1,264_ = 1.015, *P *=* *0.315) nor did fungal treatment affect adult size (*F*_1,18_ = 0.372, *P *=* *0.550) (Fig. 3B). There was no interaction between development time and the fungal treatment (*F*_1,264_ = 0.516, *P *=* *0.473).

Development time of brood beetles was highly variable (GLM model parameter estimate for development time of natal beetles = 151.49 (±15.15) days and non‐natal beetles = 168.15 (±23.68) days). There was no significant difference in development times of either sex (*F*_1,522_ = 0.060, *P *=* *0.807). Fungal treatment (natal or non‐natal) also did not significantly influence development time (*F*_1,19_ = 0.332, *P *=* *0.571), and there was no interaction between the development time of either sex and fungal treatment (*F*_1,522_ = 0.293, *P *=* *0.589).

## Discussion

Many insects rely on mutualistic symbionts for nutritional supplementation (Mueller et al. 2005; Moran 2007). Experimentally testing the obligate nature of the mutualism is an important first step in understanding these types of symbiosis. Further, verifying the level of reciprocal adaptation between the host and symbiont is crucial for establishing the strength of coevolution between species (Mueller 2012). Here, we demonstrated a requirement of symbiotic fungi, specifically *E*. sp. B, for supporting growth and development of western pine beetle. We also found that the fungal isolate/haplotype obtained from western pine beetle from the southwest (i.e., the other cryptic beetle species) was capable of supporting development of beetles from Montana. However, our most striking finding was the inability of brood adult beetles from Montana to acquire the southwest isolate/haplotype in their mycangia. Our results suggest that western pine beetles are adapted to particular fungal partners and that fidelity may be enforced through mycangial selectivity.

We observed a near‐complete loss of brood production when beetles were reared without a fungal symbiont. Our results are similar to studies that have been conducted on the closely related southern pine beetle (*Dendroctonus frontalis* Zimmerman) system, where there is a strong negative effect on beetles when they are reared without their mycangial fungi (*Entomocorticium* sp. A and *C. ranaculosus*). Barras (1973) experimentally manipulated the southern pine beetle symbiosis and found that nonmanipulated adult beetles produced nearly triple (2.93×) the number of adult offspring compared to beetles that were surface‐sterilized as pupae. Goldhammer et al. (1990) conducted laboratory experiments with beetles naturally with and without mycangial fungi and found that beetles without mycangial fungi were able to produce brood adults. Unfortunately, the number of offspring per pairing was not quantified in the experiment. Goldhammer et al. (1990) did find that brood adults that developed without fungi were much smaller, unable to reproduce, and unsuccessful at initiating galleries. Our methods for testing the importance of the mycangial fungi differed from those of Barras (1973) and Goldhammer et al. (1990) in that we subjected all pupae to surface sterilization and then re‐established the symbiosis with only one symbiont. Our methods, thereby, isolated the effect of only one factor, *E*. sp. B, on beetle reproduction and eliminated surface sterilization as a confounding factor. In both Barras (1973) and Goldhammer et al. (1990), yeasts and bacteria remaining on the exoskeleton or in the mycangia may have influenced results.

In our study, we found no evidence of a reduction in parent gallery length or of reduced oviposition in our no fungus treatment, suggesting little direct impact on reproductive investment from aposymbiotic parent adults. Our results are consistent with Barras (1973) who also failed to find differences in reproductive input from surface‐sterilized southern pine beetles. However, our results and those of Barras (1973) are in contrast to Goldhammer et al.(1990), who identified decreases in gallery production and oviposition of southern pine beetles without mycangial fungi relative to those with fungi. The differences between the studies warrant further research into the relative importance of the mycangial fungi to different bark beetle life stages.

Comparisons of brood production of beetles reared on the two different haplotypes of *E*. sp. B (i.e., natal and non‐natal) in our study suggest that both are equally capable of providing the nutritional requirements of the host beetle. We observed no differences between natal and non‐natal treatments with regard to total number of brood adults, their size, or their development time, despite the fact that the two fungal isolates are genetically divergent (Bracewell and Six 2014) and may have been geographically isolated, along with their hosts, for a long period of time (Kelley et al. 1999). This may indicate strong selection to maintain characteristics in the fungi that provide appropriate nutrients to the host and that these requirements do not differ between the two cryptic species of beetle. This may be a general feature of mycangial fungi as a fungal swap experiment in ambrosia beetles (bark beetles that are dependent on mycangial fungi) also suggests little impact on reproductive output when beetles were reared with a different mycangial fungus (Kaneko and Takagi 1966).

However, while the fungi did not differ in their effects on beetle development and productivity, the Montana beetles never acquired the southwestern fungus in their mycangia and the resulting brood females were aposymbiotic. This implies that beetles may have diverged along with their fungi and that they are adapted to particular genotypes of symbionts enforcing a high degree of specificity and fidelity at a very fine scale. Although our experimental design left us unable to quantify fungal growth in each treatment or confirm fungal sporulation of the non‐natal isolate in the pupal chamber (necessary for incorporation in the mycangia), our results do not suggest that the non‐natal fungus grew or sporulated differently than the natal fungus. Had the non‐natal fungus grew poorly or not sporulated, brood adults would have likely been smaller (e.g., Goldhammer et al. 1990) and development times would have likely been significantly different between treatments. Many bark beetle species maturation feed on fungal spores in the pupal chamber and fungal absence can lead to delayed emergence. It is important to note that due to limitations of our experiment, only one representative isolate per haplotype was tested. Although our results likely apply broadly to each fungus, we cannot rule out isolate‐specific effects influencing our results. Future work should examine isolate‐specific differences and how they might impact the symbiosis in general as recent work suggests some variation in growth rate of *Entomocorticium* isolates (Dysthe et al. 2015). Further, an experimental manipulation of the western pine beetle symbiosis that tests whether beetles from the southwest can incorporate haplotype A into their mycangia will help determine whether our observed pattern of specificity is reciprocal.

There are a number of species in the bark beetle genus *Dendroctonus* that possess mycangia (Six and Klepzig 2004). Many of these species overlap in distribution and in host tree species range (Wood 1982) and are often found co‐inhabiting the same tree. This means that, although symbiont transfer from parent to offspring is thought to primarily occur via vertical transfer, beetles that co‐occur in host trees are exposed to a large pool of potential fungal symbionts that occur due to the presence of congeneric beetles. Regardless of spatial and temporal overlap, these bark beetles still exhibit high fidelity with their fungal partners. Research to date suggests that, among mycangium‐bearing *Dendroctonus*, there is remarkable fidelity of particular fungi with their host beetles and no evidence of swapping (although, in some cases, additional partners have been acquired) (Six and Paine 1997; Alamouti et al. 2011; Roe et al. 2011; Bracewell and Six 2014).

How the beetle maintains these associations through time is not well understood, but our results indicate mycangia may play a key role enforcing specificity. Bark beetle mycangia have complex morphologies and contain glands which secrete substances thought to nurture fungi during transport (Happ et al. 1971; Bleiker et al. 2009; Yuceer et al. 2011). Given the importance of these structures in maintaining the symbiosis through consistent dissemination between generations of beetles, it is plausible the structure and its glandular secretions may play a primary role in filtering fungal species and promoting associations with specific beneficial symbiotic partners. Future research aimed at identifying the substance(s) being excreted by the mycangia and additional fungal swapping experiments in other bark beetles will go a long way in understanding the ability of the mycangia to screen fungal symbionts and whether glandular excretions may be fine‐tuned to promote particular associations.

For insects involved in obligate mutualisms with microbes, maintaining associations with particular partners is crucial for the success and stability of the symbiosis. However, currently, we have only a rudimentary understanding of the mechanisms that underlie fidelity or evolutionary processes in ectosymbioses, including bark beetle–fungus symbioses. We have shown in other work that the western pine beetle symbiosis exhibits high fidelity (Bracewell and Six 2014). In the research reported in this study, we have shown through experimental manipulation of the symbiosis that fungi are critical to the beetle's survival. Further, our results suggest that the beetle's mycangia can distinguish between closely related fungal isolates and may play a key role in maintaining specificity. This, in turn, indicates adaptation of the beetle at a fine scale to its fungal partners. Additional research is needed to reveal whether fidelity and coevolution are common in bark beetle–fungus symbioses and what conditions facilitate or constrain coevolution and codiversification.

## Conflict of Interest

None declared.

## Appendix 1

**Table:**

Treatment	Replicate	Total brood	Total females	Isolated	Positive for natal fungus^a^	Isolated females positive for natal fungus (%)
Natal	3	55	26	8	4	50
Natal	5	37	14	6	5	83
Natal	6	28	12	6	4	67
Natal	8	18	12	4	3	75
Natal	10	48	18	10	8	80
Natal	11	6	1	1	1	100
Natal	12	44	16	4	0	0
Natal	13	8	4	3	2	67
Natal	14	77	36	4	4	100
Natal	15	1	1	1	0	0
Natal	16	3	2	2	0	0
Non‐natal	2	10	4	4	0	0
Non‐natal	3	8	4	4	3	75
Non‐natal	6	22	3	3	0	0
Non‐natal	7	31	20	14	0	0
Non‐natal	8	30	15	14	0	0
Non‐natal	10	25	13	10	0	0
Non‐natal	13	15	12	11	0	0
Non‐natal	15	112	20	6	4	67
Non‐natal	16	66	40	9	0	0
Non‐natal	17	51	28	6	4	67
No fungus	2	1	1	0	0	NA
No fungus	7	2	2	1	0	0
No fungus	16	29	11	5	4	80

Note

a Identified by sequencing ITS2‐LSU region of representative isolate and morphotyping remaining isolates.

## References

[ece31772-bib-0001] Alamouti, S. M. , V. Wang , S. Diguistini , D. L. Six , J. Bohlmann , R. C. Hamelin , et al. 2011 Gene genealogies reveal cryptic species and host preferences for the pine fungal pathogen *Grosmannia clavigera* . Mol. Ecol. 20:2581–2602. 21557782 10.1111/j.1365-294X.2011.05109.x

[ece31772-bib-0002] Amman, G. D. 1972 Mountain pine beetle brood production in relation to thickness of lodgepole pine phloem. J. Econ. Entomol. 65:138–140.

[ece31772-bib-0003] Anderbrant, O. 1990 Gallery construction and oviposition of the bark beetle *Ips typographus* (Coleoptera, Scolytidae) at different breeding densities. Ecol. Entomol. 15:1–8.

[ece31772-bib-0004] Ayres, M. P. , R. T. Wilkens , J. J. Ruel , M. J. Lombardero , and E. Vallery . 2000 Nitrogen budgets of phloem‐feeding bark beetles with and without symbiotic fungi. Ecology 81:2198–2210.

[ece31772-bib-0005] Barras, S. J. 1973 Reduction of progeny and development in southern pine beetle following removal of symbiotic Fungi. Can. Entomol. 105:1295–1299.

[ece31772-bib-0006] Bentz, B. J. , J. A. Logan , and J. C. Vandygriff . 2001 Latitudinal variation in *Dendroctonus ponderosae* (Coleoptera: Scolytidae) development time and adult size. Can. Entomol. 133:375–387.

[ece31772-bib-0007] Betancourt, J. L. , T. R. Van Devender , and P. S. Martin . 1990 Packrat middens: the last 40,000 years of biotic change. University of Arizona Press, Tucson, AZ. 10.1126/science.250.4983.1021-a 17746928

[ece31772-bib-0008] Bleiker, K. P. , and D. L. Six . 2007 Dietary benefits of fungal associates to an eruptive herbivore: potential implications of multiple associates on host population dynamics. Environ. Entomol. 36:1384–1396. 18284766 10.1603/0046-225x(2007)36[1384:dbofat]2.0.co;2

[ece31772-bib-0009] Bleiker, K. P. , S. E. Potter , C. R. Lauzon , and D. L. Six . 2009 Transport of fungal symbionts by mountain pine beetles. Can. Entomol. 141:503–514.

[ece31772-bib-0010] Bracewell, R. R. , and D. L. Six . 2014 Broadscale specificity in a bark beetle‐fungal symbiosis: a spatio‐temporal analysis of the mycangial fungi of the western pine beetle. Microb. Ecol. 68:859–870. 25004995 10.1007/s00248-014-0449-7

[ece31772-bib-0011] Bracewell, R. R. , M. E. Pfrender , K. E. Mock , and B. J. Bentz . 2011 Cryptic postzygotic isolation in an eruptive species of bark beetle (*Dendroctonus ponderosae*). Evolution 65:961–975. 21108639 10.1111/j.1558-5646.2010.01201.x

[ece31772-bib-0012] Clark, M. A. , N. A. Moran , P. Baumann , and J. J. Wernegreen . 2000 Cospeciation between bacterial endosymbionts (*Buchnera*) and a recent radiation of aphids (*Uroleucon*) and pitfalls of testing for phylogenetic congruence. Evolution 54:517–525. 10937228 10.1111/j.0014-3820.2000.tb00054.x

[ece31772-bib-0013] Conkle, M. T. , and W. B. Critchfield . 1988 Genetic variation and hybridization of ponderosa pine Pp. 27–43 *in* Baumgartner D. M. and Lotan J. E., eds. Ponderosa pine: the species and its management. Washington State Univ. Press, Pullman, WA.

[ece31772-bib-0014] Conord, C. , L. Despres , A. Vallier , S. Balmand , C. Miquel , S. Zundel , et al. 2008 Long‐term evolutionary stability of bacterial endosymbiosis in curculionoidea: additional evidence of symbiont replacement in the dryophthoridae family. Mol. Biol. Evol. 25:859–868. 18310662 10.1093/molbev/msn027

[ece31772-bib-0015] Coppedge, B. R. , F. M. Stephen , and G. W. Felton . 1995 Variation in female southern pine beetle size and lipid content in relation to fungal associates. Can. Entomol. 127:145–154.

[ece31772-bib-0016] Dysthe, J. C. , R. Bracewell , and D. L. Six . 2015 Temperature effects on growth of fungal symbionts of the western pine beetle, *Dendroctonus brevicomis* . Fung. Ecol. 17:62–68.

[ece31772-bib-0017] de Fine Licht, H. H. , J. J. Boomsma , and D. K. Aanen . 2007 Asymmetric interaction specificity between two sympatric termites and their fungal symbionts. Ecol. Entomol. 32:76–81.

[ece31772-bib-0018] Foelker, C. J. , and R. W. Hofstetter . 2014 Heritability, fecundity, and sexual size dimorphism in four species of bark beetles (Coleoptera: Curculionidae: Scolytinae). Ann. Entomol. Soc. Am. 107:143–151.

[ece31772-bib-0019] Goldhammer, D. S. , F. M. Stephen , and T. D. Paine . 1990 The effect of the fungi *Ceratocystis minor* (Hedgecock) Hunt, *Ceratocystis minor* (Hedgecock) Hunt Var. *Barrasii* Taylor, and SJB 122 on reproduction of the southern pine beetle, *Dendroctonus frontalis* Zimmermann (Coleoptera, Scolytidae). Can. Entomol. 122:407–418.

[ece31772-bib-0020] Happ, G. M. , C. M. Happ , and S. J. Barras . 1971 Fine structure of prothoracic mycangium, a chamber for culture of symbiotic fungi, in southern pine beetle, *Dendroctonus frontalis* . Tissue Cell 3:295–308. 18631556 10.1016/s0040-8166(71)80024-1

[ece31772-bib-0021] Harrington, T. C. 2005 Ecology and evolution of mycophagous bark beetles and their fungal partners Pp. 257–291 *in* Vega F. E., Blackwell M., eds. Ecological and evolutionary advances in insect‐fungal associations. Oxford Univ. Press, New York.

[ece31772-bib-0022] Honek, A. 1993 Intraspecific variation in body size and fecundity in insects: a general relationship. Oikos 66:483–492.

[ece31772-bib-0023] Hothorn, T. , F. Bretz , P. Westfall , R. M. Heiberger , and A. Schuetzenmeister , 2014 multcomp: simultaneous inference in general parametric models, Version 1.3‐1. 10.1002/bimj.200810425 18481363

[ece31772-bib-0024] Hsiau, P. T. W. , and T. C. Harrington . 1997 *Ceratocystiopsis brevicomi* sp. nov., a mycangial fungus from *Dendroctonus brevicomis* (Coleoptera: Scolytidae). Mycologia 89:661–669.

[ece31772-bib-0025] Hsiau, P. T. W. , and T. C. Harrington . 2003 Phylogenetics and adaptations of basidiomycetous fungi fed upon by bark beetles (Coleoptera: Scolytidae). Symbiosis 34:111–131.

[ece31772-bib-0026] Jacobs, K. , and M. J. Wingfield . 2001 Leptographium species: tree pathogens, insect associates, and agents of blue‐stain. The American Phytopathological Society, St. Paul, MN.

[ece31772-bib-0027] Kaneko, T. , and K. Takagi . 1966 Biology of some scolytid ambrosia beetles attacking tea plants. VI. A comparative study of two ambrosia fungi associated with *Xyleborus compactus* Eichhoff and *Xyleborus germanus* Blandford (Coleoptera: Scolytidae). Appl. Ent. Zool. 4:173–176.

[ece31772-bib-0028] Kelley, S. T. , J. B. Mitton , and T. D. Paine . 1999 Strong differentiation in mitochondrial DNA of *Dendroctonus brevicomis* (Coleoptera: Scolytidae) on different subspecies of ponderosa pine. Ann. Entomol. Soc. Am. 92:193–197.

[ece31772-bib-0029] Latta, R. G. , and J. B. Mitton . 1999 Historical separation and present gene flow through a zone of secondary contact in ponderosa pine. Evolution 53:769–776. 10.1111/j.1558-5646.1999.tb05371.x 28565634

[ece31772-bib-0030] Mehdiabadi, N. J. , U. G. Mueller , S. G. Brady , A. G. Himler , and T. R. Schultz . 2012 Symbiont fidelity and the origin of species in fungus‐growing ants. Nat. Commun., 3, 840. 22588302 10.1038/ncomms1844

[ece31772-bib-0031] Miller, J. M. , and F. P. Keen . 1960 Biology and control of the western pine beetle: a summary of the first 50 years of research. U.S. Department of Agriculture, Washington, DC.

[ece31772-bib-0032] Moran, N. A. 2007 Symbiosis as an adaptive process and source of phenotypic complexity. PNAS 104:8627–8633. 17494762 10.1073/pnas.0611659104 PMC1876439

[ece31772-bib-0033] Moran, N. A. , and G. M. Bennett . 2014 The tiniest tiny genomes. Annu. Rev. Microbiol. 68:195–215. 24995872 10.1146/annurev-micro-091213-112901

[ece31772-bib-0034] Mueller, U. G. 2012 Symbiont recruitment versus ant‐symbiont co‐evolution in the attine ant‐microbe symbiosis. Curr. Opin. Microbiol. 15:269–277. 22445196 10.1016/j.mib.2012.03.001

[ece31772-bib-0035] Mueller, U. G. , N. M. Gerardo , D. K. Aanen , D. L. Six , and T. R. Schultz . 2005 The evolution of agriculture in insects. Annu. Rev. Ecol. Evol. Syst. 36:563–595.

[ece31772-bib-0036] Paine, T. D. , and M. C. Birch . 1983 Acquisition and maintenance of mycangial fungi by *Dendroctonus brevicomis* Leconte (Coleoptera, Scolytidae). Environ. Entomol. 12:1384–1386.

[ece31772-bib-0037] Paine, T. D. , K. F. Raffa , and T. C. Harrington . 1997 Interactions among scolytid bark beetles, their associated fungi, and live host conifers. Annu. Rev. Entomol. 42:179–206. 15012312 10.1146/annurev.ento.42.1.179

[ece31772-bib-0038] Pinheiro, J. , D. Bates , S. DebRoy , and D. Sarkar , 2015 Package ‘nlme’.

[ece31772-bib-0039] Potter, K. , V. Hipkins , M. Mahalovich , and R. Means . 2015 Nuclear genetic variation across the range of ponderosa pine (*Pinus ponderosa*): phylogeographic, taxonomic and conservation implications. Tree Genet. Genomes 11:1–23.

[ece31772-bib-0500] R Development Core Team . 2011 R: A Language and Environment for Statistical Computing. R Foundation for Statistical Computing, Vienna. Available at http://www.R-project.org.

[ece31772-bib-0040] Roe, A. D. , A. V. Rice , D. W. Coltman , J. E. K. Cooke , and F. A. H. Sperling . 2011 Comparative phylogeography, genetic differentiation and contrasting reproductive modes in three fungal symbionts of a multipartite bark beetle symbiosis. Mol. Ecol. 20:584–600. 21166729 10.1111/j.1365-294X.2010.04953.x

[ece31772-bib-0041] Seal, J. N. , and U. G. Mueller . 2014 Instability of novel ant‐fungal associations constrains horizontal exchange of fungal symbionts. Evol. Ecol. 28:157–176.

[ece31772-bib-0042] Six, D. L. 2012 Ecological and evolutionary determinants of bark beetle—fungus symbioses. Insects 3:339–366. 26467964 10.3390/insects3010339 PMC4553632

[ece31772-bib-0043] Six, D. L. , and K. D. Klepzig . 2004 *Dendroctonus* bark beetles as model systems for studies on symbiosis. Symbiosis 37:207–232.

[ece31772-bib-0044] Six, D. L. , and T. D. Paine . 1997 *Ophiostoma clavigerum* is the mycangial fungus of the Jeffrey pine beetle, *Dendroctonus jeffreyi* . Mycologia 89:858–866.

[ece31772-bib-0045] Six, D. L. , and T. D. Paine . 1999 Phylogenetic comparison of ascomycete mycangial fungi and *Dendroctonus* bark beetles (Coleoptera: Scolytidae). Ann. Entomol. Soc. Am. 92:159–166.

[ece31772-bib-0046] Upadhyay, H. P. 1981 A monograph of ceratocystis and ceratocystiopsis. The University of Georgia Press, Athens, GE.

[ece31772-bib-0047] Valiev, A. , Z. B. Ogel , and K. D. Klepzig . 2009 Analysis of cellulase and polyphenol oxidase production by southern pine beetle associated fungi. Symbiosis 49:37–42.

[ece31772-bib-0048] Vilgalys, R. , and M. Hester . 1990 Rapid genetic identification and mapping of enzymatically amplified ribosomal DNA from several *Cryptococcus* Species. J. Bacteriol. 172:4238–4246. 2376561 10.1128/jb.172.8.4238-4246.1990 PMC213247

[ece31772-bib-0049] White, T. J. , T. Bruns , S. Lee , and J. W. Taylor . 1990 Amplification and direct sequencing of fungal ribosomal RNA genes for phylogenetics Pp. 315–322 *in* Innis M. D., Gelfand D. H., Sninsky J. J. and White T. J., eds. PCR protocols: a guide to methods and applications. Academic Press Inc, New York.

[ece31772-bib-0050] Whitney, H. S. , and F. W. Cobb . 1972 Non‐staining fungi associated with the bark beetle *Dendroctonus brevicomis* (Coleoptera: Scolytidae) on *Pinus ponderosa* . Can. J. Bot. 50:1943–1945.

[ece31772-bib-0051] Wood, S. L. 1982 The bark and ambrosia beetles of North and Central America (Coleoptera: Scolytidae). Great Basin Nat. 6:1–1359.

[ece31772-bib-0052] Yuceer, C. , C. Y. Hsu , N. Erbilgin , and K. D. Klepzig . 2011 Ultrastructure of the mycangium of the southern pine beetle, *Dendroctonus frontalis* (Coleoptera: Curculionidae, Scolytinae): complex morphology for complex interactions. Acta Zool. 92:216–224.

